# Development and validation of whole genome-wide and genic microsatellite markers in oil palm (*Elaeis guineensis* Jacq.): First microsatellite database (OpSatdb)

**DOI:** 10.1038/s41598-018-37737-7

**Published:** 2019-02-13

**Authors:** Kalyana Babu B., Mary Rani K. L., Sarika Sahu, R. K. Mathur, Naveen Kumar P., Ravichandran G., Anitha P., Bhagya H. P.

**Affiliations:** 1 ICAR-Indian Institute of Oil Palm Research, Pedavegi-534 450, West Godavari (Dt), Andhra Pradesh, India; 2 ICAR-Indian Agricultural Statistics Research Institute, New Delhi, 12 India

## Abstract

The availability of large expressed sequence tag (EST) and whole genome databases of oil palm enabled the development of a data base of microsatellite markers. For this purpose, an EST database consisting of 40,979 EST sequences spanning 27 Mb and a chromosome-wise whole genome databases were downloaded. A total of 3,950 primer pairs were identified and developed from EST sequences. The tri and tetra nucleotide repeat motifs were most prevalent (each 24.75%) followed by di-nucleotide repeat motifs. Whole genome-wide analysis found a total of 245,654 SSR repeats across the 16 chromosomes of oil palm, of which 38,717 were compound microsatellite repeats. A web application, OpSatdb, the first microsatellite database of oil palm, was developed using the PHP and MySQL database (https://ssr.icar.gov.in/index.php). It is a simple and systematic web-based search engine for searching SSRs based on repeat motif type, repeat type, and primer details. High synteny was observed between oil palm and rice genomes. The mapping of ESTs having SSRs by Blast2GO resulted in the identification of 19.2% sequences with gene ontology (GO) annotations. Randomly, a set of ten genic SSRs and five genomic SSRs were used for validation and genetic diversity on 100 genotypes belonging to the world oil palm genetic resources. The grouping pattern was observed to be broadly in accordance with the geographical origin of the genotypes. The identified genic and genome-wide SSRs can be effectively useful for various genomic applications of oil palm, such as genetic diversity, linkage map construction, mapping of QTLs, marker-assisted selection, and comparative population studies.

## Introduction

Oil palm (*Elaeis guineensis* Jacq.) is a perennial crop, belonging to the family Arecaceae, and is the major source of edible vegetable oil of the world (53.3 Mt followed by Soybean oil, 43.4 Mt). It consists of two species *viz*., African oil palm (*Elaeis guineensis*) and American oil palm (*E. oleifera*). Although the crop originated in Africa, two South East Asian countries (Malaysia and Indonesia) account for more than 50% of the world’s oil palm plantations^1^. Oil palm has 16 pairs of chromosomes, with a genome size of 1.8 Gb. The full draft genome sequence of 1.535 Gb of *E. guineensis* was recently published^2^ and is freely available. Oil palm is a tropical crop, with a theoretical potential oil yield capacity of 10 t/ha, however, current trends are far below the potential oil yields, which vary between 2–6 t/ha of oil^3^. To further increase the oil yields, there is a great need to develop molecular markers for marker-assisted breeding programs to facilitate genetic improvement in yield, oil quality and other important agro-morphological traits of interest.

Microsatellite or simple sequence repeat (SSR) markers are widely used markers of choice by many plant breeders and molecular biologists because of their high levels of polymorphism, co-dominant inheritance, and reproducibility, as well as their applicability for genetic diversity, linkage mapping, marker-trait associations and marker-assisted selection programs. The SSR markers have been used in oil palm for various purposes *viz*., genetic diversity^4^, and construction of linkage maps^5^, QTL mapping^6^, and association mapping^7^. In the case of oil palm, a few hundred SSRs are available in the public domain^5^, which are not sufficient for fine mapping and genetic diversity studies. However, development of SSRs is often tedious; cloning and enrichment procedures are required for their generation and are costly^8^. The expressed sequence tag (EST)- based SSRs within the genic regions are more transferable to closely related species, as they represent conserved genic regions of chromosomes. The availability of large data sets of the EST database have become attractive resources for *in silico* studies, as was demonstrated in cereals^9^, date palm^10^, and coco nut^11^. In the case of oil palm, few reports^12^ are available on the identification of EST- based SSRs. However, all of these reports are based on few EST sequences. Singh *et al*.^13^ exploited 5,521 EST sequences and found 145 SSRs in 136 unique ESTs, and few of them were used for genetic diversity studies. Ting *et al*.^12^ mined 19,243 *oil palm* ESTs and found 10,258 unique sequences, of which 629 ESTs were found to contain 722 SSRs with a variety of motifs. However, until December 1, 2017, a total of 40,979 EST sequences of oil palm were available in the NCBI website. Genome-wide SSRs play a very important role in genomics applications; however, very few SSRs of oil palm are available in the public domain. *In silico* characterization of genomic SSRs has been exploited in a few crops, such as fox tail millet^14^ and tomato^15^. Until now, very few reports have been available on crop-based microsatellite databases^15^; however in oil palm no such database is available. With this aim, the objectives of present study are (1) *in silico* mining of genic and whole genome-wide microsatellites of oil palm, along with their frequency and distribution analysis; (2) validation, polymorphism and genetic diversity analysis of genic and genome-wide SSR markers among 100 oil palm genetic resources belongs to 18 accessions; (3) functional annotation of the EST sequences; and (4) design and development of a web application microsatellite database of oil palm.

## Results and Discussion

### Frequency and distribution of genic SSRs in the oil palm genome

The present study is the first report on the identification of EST-based SSRs (EST-SSRs) using a large number of EST sequences available in the database of oil palm. A total of 40,979 EST sequences, representing approximately 27 Mb of oil palm genome, were downloaded in fasta format and searched for microsatellites. A total of 3,950 primer pairs were developed from EST sequences, where in one microsatellite marker occurred for every 6.7 Kb of EST sequences. In other words 138 SSRs were identified for one mega base of oil palm genome. The number of SSRs obtained in the present investigation was higher than the earlier report in oil palm^16^, where they found one SSR for every 5.7 Kb of EST sequence, and lower than a few reports in oil palm^12^. The results were also similar to other crops; for example, in the *Arabidopsis* genome, 127.5 SSRs/Mb were obtained, however but oil palm was less than the rice genome, where 189.4 SSRs/Mb was found and greater than the sorghum database (99.8 SSRs/Mb)^17^ and foxtail millet (69 SSRs/Mb)^14^. These variations could be due to different search criteria, sizes of databases and software tools used for identification of SSRs.

The SSRs identified consisted of 1000 mono-, 500 di-, 195 tri-, 924 tetra-, 132 penta- and 245 hexa-nucleotide repeat motifs (Table 1). Out of the 3,950 microsatellites, excluding mono-nucleotide repeats (MNRs), di-nucleotide repeats (DNRs) (31.1%) were the predominant SSR, followed by tri-nucleotide repeats (TNRs) (5.9%), tetra-nucleotide repeats (TeNRs) (0.75%) and further followed by hexa-nucleotide repeats (HNRs) and penta-nucleotide repeats (PNRs) (The minimum number of repeats considered was 6 for percentage calculation) (Supplemental Figure S1). Similarly, in the earlier reports of oil palm, DNRs were found to be more frequent SSR repeat motifs, followed by TNRs^16^. Generally, DNRs are the most frequent, as in the case of Arabidopsis, rice and sorghum^18^; however, in several crops TNRs are the predominant repeat motifs as in Brachypodium^17^, foxtail millet^14^, bamboo^19^, switch grass, and coconut^20^. Among the TNRs, AAG/CTT was most frequent (8%), followed by AGG/CCT (5%) and CCG/CGG (4%). Among the DNRs, AG/CT represented the most frequent form (43%), followed by AT/TA (20.3%), AC/GT (2.8%) and GC/CG (0.8%) (Table 1). The results obtained were similar to the earlier report on oil palm by Ting *et al*.^12^, where they also found the same frequency of repeat motifs in the order of TNRs and DNRs. The AG/CT DNRs were also the most frequent repeat types in most of the cereals^18^, millets^21^ and other crops^16^. Among the MNRs, A/T represented about 89%, followed by C/G repeats, which represented 11% of the total MNRs.

**Table 1:** Details of SSR repeat motifs (MNRs, DNRs, TNRs, TeNRs, PNRs, and HNRs) among the EST sequences of oil palm. The table represents the number of SSRs identified for each category of repeat motif.

SSR motifs	Number of repeats																		Total
	3	4	5	6	7	8	9	10	11	12	13	14	15	16	17	18	19	>=20	
MNRs	—	—	—	4	1	1	0	309	161	87	86	63	59	34	40	67	31	57	1000
DNRs	—	—	—	128	52	80	98	33	21	13	8	10	57 (>15)	—	—	—	—	—	500
TNRs	—	—	101	62	14	13	2	2	1	0	0	0	0	—	—	—	—	—	195
TeNRs	779	105	28	07	0	01	03	01	—	—	—	—	—	—	—	—	—	—	924
PNRs	115	12	05	0	0	0	0	0	—	—	—	—	—	—	—	—	—	—	132
HNRs	210	31	03	01	0	0	0	0	—	—	—	—	—	—	—	—	—	—	245
A/T				3	1	1	0	255	136	74	79	56	55	31	39	66	30	57	883
C/G				1	0	0	0	54	25	13	7	7	4	3	1	1	1	0	117
AG/CT				85	31	39	68	21	18	7	4	9							282
AC/GT				8	7	1	1	1	0	0	2	1							21
AT/TA				31	14	39	29	11	3	6	2	0							135
GC/CG				4	0	1	0	0	0	0	0	0							5
AAG/CTT			36	31	4	5	1	0	1	0	0	0							78
AGG/CCT			29	14	4	4	1	0	0	0	0	0							52
CCG/CGG			20	9	6	4	0	0	0	0	0	0							39
AAC/GTT			1	2	0	0	0	2	0	0	0	0							5
ACC/GGT			15	6	0	0	0	0	0	0	0	0							21

### Frequency and distribution of whole genome-wide SSRs in the oil palm genome

**Figure 1:**
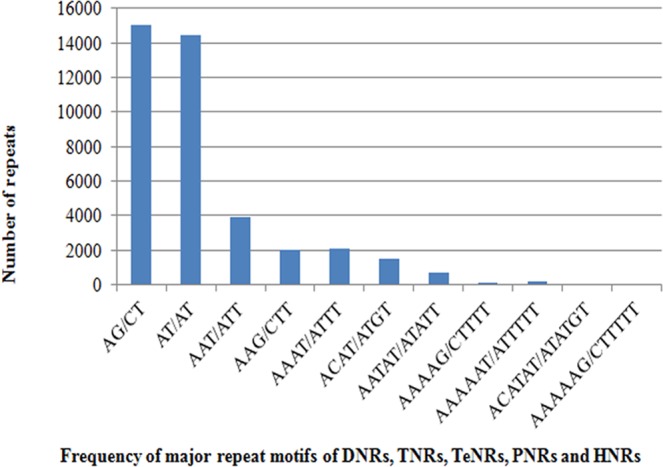
The frequency of major repeat motifs of DNRs, TNRs, TeNRs, PNRs and HNRs across the sixteen chromosomes of oil palm.

Whole genome sequence of oil palm was downloaded from the NCBI website separately for all 16 chromosomes. The density of microsatellite repeats observed in descending order was 584, 391, 388, and 380 per Mb in chromosomes 1, 12, 10 and 16 respectively. A total of 245,654 microsatellite repeats were found across the 16 chromosomes. Chromosome 1 had more SSR repeats (39,987), followed by chromosomes 2 (24,032) and 3 (21,731) (Table 2). A similar pattern was also observed for compound microsatellites, where chromosome 1 had more compound microsatellites, followed by chromosomes 2 and 3. The least SSR repeats were observed in chromosome 16 (8,111), whereas the least compound microsatellites were observed in chromosome 15 (1,232). DNRs (67,570) were the most frequent across the chromosomes, followed by TNR motifs (18,353). Similar reports were obtained in oil palm by Xiao *et al*.^22^. The graphical representation of the distribution of DNRs, TNRs, TeNRs, PNRs and HNRs across the 16 chromosomes is given in Fig. 1. In the case of chromosome 1, the most frequent repeats are DNRs, followed by TeNRs and TNRs. However, in the remaining chromosomes, DNRs are most frequent, followed by TNRs, TeNRs, PNRs and HNRs. Among the DNRs, AG/CT (15,050) motifs are abundant, followed by AT/AT repeat motifs (14,451). In the case of TNRs, AAT/ATT motifs are the most frequent, followed by AAG/CTT repeat motifs (Fig. 1). Xiao *et al*.^22^ identified genome-wide SSRs in oil palm, but the present study gives an elaborate analysis of genic SSRs in addition to genome-wide SSRs. Also, the synteny between oil palm and rice chromosomes was studied, and the first ever web application for a microsatellite database in oil palm was developed. A total of 187,980 primer pairs were designed across the 16 chromosomes of oil palm. Chromosome 1 was found to have more primer pairs (22,536), followed by chromosome 2 (20,410) (Table 2). An average of 366 SSR primers/ Mb was designed among the 16 chromosomes.

**Table 2:** The total number of SSRs and compound SSRs of oil palm by chromosome. The table also denotes identified SSR primers of DNRs, TNRs, TeNRs, PNRs and HNRs, as well as number of SSRs per Mb of genome sequence for each chromosome.

Chromosome	Total number of SSRs	Compound SSRs	Di-repeats	Tri-repeats	Tetra-repeats	Penta-repeats	Hexa-repeats	SSR primers	SSR/Mb
1	39,987	7,589	7,697	5,031	6,953	2,432	1,137	22,536	584
2	24,032	3,623	7,001	1,544	484	88	34	20,410	367
3	21,731	3,372	6,229	1,290	445	78	40	18,360	362
4	18,790	2,707	5,531	1,239	372	78	28	16,084	328
5	17,963	2,669	5,247	1,166	359	83	17	15,295	346
6	13,275	1,860	3,736	923	248	53	18	11,416	299
7	15,146	2,327	4,359	985	344	58	24	12,820	349
8	14,412	2,282	4,166	980	335	76	21	12,131	359
9	11,254	1,695	3,388	734	209	59	12	9,560	296
10	12,361	1,992	3,729	772	243	51	27	10,370	388
11	9,834	1,375	2,755	642	194	47	8	8,460	327
12	11,265	1,805	3,294	726	225	44	22	9,461	391
13	10,038	1,531	2,978	622	211	49	19	8,508	361
14	8,900	1,374	2,523	565	189	30	16	7,527	365
15	8,555	1,232	2,503	585	154	29	16	2,521	352
16	8,111	1,284	2,434	549	173	27	13	2,521	380
Total	245,654	38,717	67,570	18,353	11,138	3,282	1,452	187,980	366

### Functional annotation of *E. guineensis* ESTs containing SSRs

The Blast2GO annotation process of oil palm EST sequences (600) containing SSRs was done in a three step process *viz*., Blastx search, mapping and annotation. The IPS results were showed for only 300 EST sequence. Of these, 40 ESTs showed blast hits and 140 ESTs showed without hits (Supplemental Figure S2). The mapping step of Blast2GO found 115 (19.2%) sequences with GO annotations and few ESTs were found with mapping. The mapping results were mainly contributed from the UniProt KB data base^23^ (90%), followed by the Arabidopsis information resource (TAIR)^24^ (8%), and GR protein (1.9%) database^25^, and a negligible amount was contributed by the protein data bank (PDB) database^26^. The GO terms’ frequency was increased proportionately with their length, such that shorter sequences had lower frequencies than longer sequences. The interpro scan (IPS)^27^ results showed that 460 (76.7%) sequences were without IPS, and 125 sequences showed IPS results, of which 60 sequences showed IPS results with GO terms. The IPS results were only available for 300 sequences (Supplemental Figure S2).

The species distribution of Blast hits found that *Elaeis guineensis*, *Phoenix dactylifera* and *Musa acuminate* subsp. *malaccensis* were the top three species in terms of hit number. However, when we filtered to the top Blast hit species distribution, *Elaeis guineensis*, *Phoenix dactylifera* and *Coffea canephora* were the top three hit species (Supplemental Figure S3). The ESTs containing microsatellite repeats were categorized into molecular function, biological processes and cell components by using combined GO graph statistics. Based on molecular function, metal ion binding elements comprised the highest score (24.3%), followed by transferase activity and protein binding gene sequences. However, in terms of biological processes, gene sequences related to stress shared a major part (21%) followed by translation and signal transduction-related sequences. In the case of cellular components, EST sequences related to integral components of membranes, followed by nucleus and cytosol, shared the top three positions. Tranbarger *et al*.^16^ found that the largest portions of ESTs were annotated with GO biological process annotations and molecular function annotations for metabolic (32% and 21%, respectively) and cellular (31% and 20%, respectively) processes. These were intra cellular (19%), containing intracellular particles (17%) or organelles (16%). Their results revealed that the most highly represented functional group was transcription and post-transcriptional regulation, followed by five ESTs with similarities to sequences involved in protein destination and storage, as well as three involved in signal transduction, cell structure and disease and defense. The Kyoto encyclopedia of genes and genomes (KEGG) maps for 29 metabolic pathways were generated.

### Validation and polymorphism analysis of genic and genome-wide SSR markers

A total of ten genic and five genome-wide SSR primers were designed and validated for their polymorphism among a set of 100 oil palm genetic resources obtained from different parts of the world. Out of ten genic SSRs, five were found to be polymorphic. Hence, these five SSRs were used for polymorphism and genetic analysis. All five genomic SSRs were found to be polymorphic across 100 oil palm genetic resources belongs to 18 accessions. The five genic SSRs yielded ten scorable alleles. The details of the primers, along with their forward and reverse primer sequences, allele number, gene diversity, heterozygosity and polymorphism information content (PIC) values, are given in Supplemental Table S4. The PIC values of all the polymorphic loci across the 100 oil palm genetic resources varied from 0.19 to 0.37, with an average of 0.30. The PIC values were found to be very low due to the low number of markers used. However the average PIC values were found to be 0.402 by earlier works^28^. Gene diversity was in the range of 0.21 to 0.49, with a mean of 0.39, which is more than in earlier reports^28^. The observed heterozygosity was in the range of 0.16 to 0.89, with an average of 0.56. In the case of genomic SSRs, the allele number, PIC, gene diversity, heterozygosity were observed in the ranges of 3- to 4, 0.09 to 0.59, 0.09 to 0.66 and 0.05 to 0.62, respectively (Supplemental Table S4). The results showed that the genomic SSRs were more polymorphic than the genic SSRs, which was confirmed by earlier works^29^.

### Genetic diversity analysis of oil palm genetic resources using genic and genome-wide SSRs

**Figure 2:**
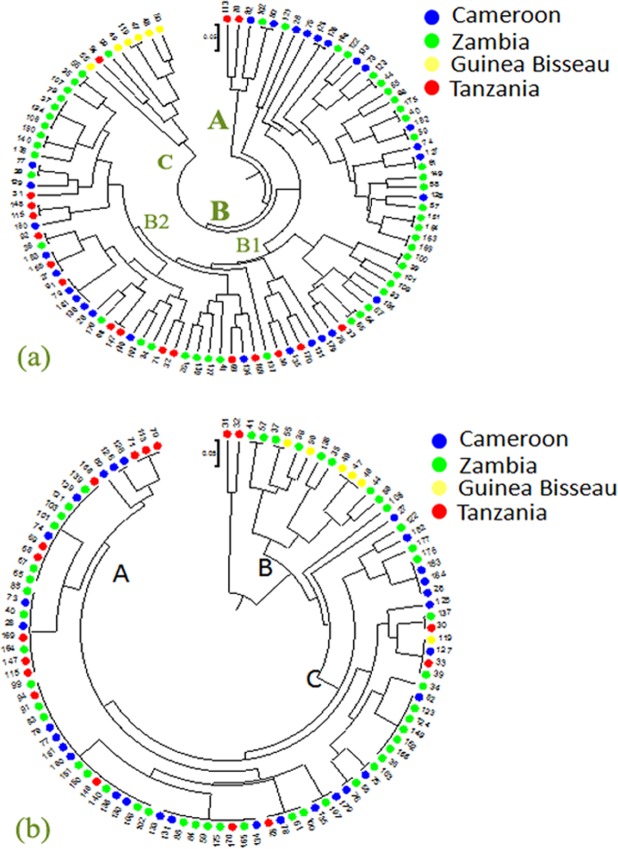
The dendrogram obtained from Power marker V3.2.5 using genome-wide (**a**) and genic (**b**) SSR markers among the 100 oil palm genetic resources.

The genome-wide SSRs grouped the 100 oil palm genetic resources belong to 18 accessions into three major groups. The grouping pattern was mostly based on their geographic origin, with few exceptions. Group A consisted of five genetic resources, with two from Tanzania (70 and 113), two from Cameroon (80 and 82) and one from Zambia (102). Group C consisted of mostly Guinea-Bissau genetic resources. Group C also consisted of two genetic resources from both Tanzania and Zambia. Group B consisted of Zambian genetic resources, which were further subdivided into two sub-groups. Sub-cluster B1 consisted of Zambia germplasm, whereas sub cluster B2 consisted of Zambian clustered together with a few Cameron and Tanzanian germplasm (Fig. 2a). Cluster C consisted of germplasm from Guinea-Bissau, Cameron and Tanzania. The genic SSRs also grouped the 100 oil palm genetic resources belong to 18 accessions into three major groups (Fig. 2b). Group A consisted of mostly Zambian and Tanzania germplasm, with a few Cameroon genetic resources, whereas group B had Guinea-Bissau and Zambian germplasm. Group C consisted of mostly Zambian and Cameroon germplasm, with few exceptions. The grouping pattern was observed to be more or less similar by both genic and genome-wide SSRs, and it would give more clear information if more SSR markers were used. The grouping pattern observed was similar to earlier reports^30^. Bakoumé *et al*.^30^ studied the extent of genetic diversity among 494 oil palms from 49 populations (representing ten African countries, three breeding materials, and one semi-wild material) using 16 SSR markers. They concluded that Madagascar populations were found to be genetically distinct from all other African populations.

### Identification of synteny and conservation among whole genome-wide chromosome sequences of oil palm and rice

The synteny and conservation between oil palm with date palm and coconut crops was already published by Mathew *et al*.^31^. They observed that most of the 18 date palm linkage groups were syntenic with one of the 16 oil palm chromosomes. Synteny determined between the date palm genetic map and the oil palm chromosomes suggests that oil palm chromosome 2 constitutes a fusion of date palm chromosomes 1 and 10. In the case of coconut, they found that most markers from each coconut linkage group aligned to the same oil palm genome. However, in the present study, the circot plot obtained between oil palm with rice using CIRCOS software^32^ includes the underlying data of oil palm and rice whole genome-wide data. Rice is a model crop and monocotyledon that may serve to identify important genes related to some useful traits such as, dwarfness. Hence, in the present study, syntenic relations were observed between oil palm and rice. It was found that chromosome 1 of rice shared homology to many chromosomes of oil palm (Supplemental Table S5). Oil palm chromosome 7 was found to be distributed among the 1^st^, 2^nd^ and 3^rd^ rice chromosomes. Oil palm chromosome 1 showed synteny with rice chromosome 1 and less synteny with chromosomes 3 and 12. The underlying data used for synteny between oil palm and rice in the study is whole genome sequence by chromosome wise for both the crops. The genomic data down loaded from NCBI website. The genomic data used as input for generating circot plot.

### Database development (OpSatdb)

**Figure 3:**
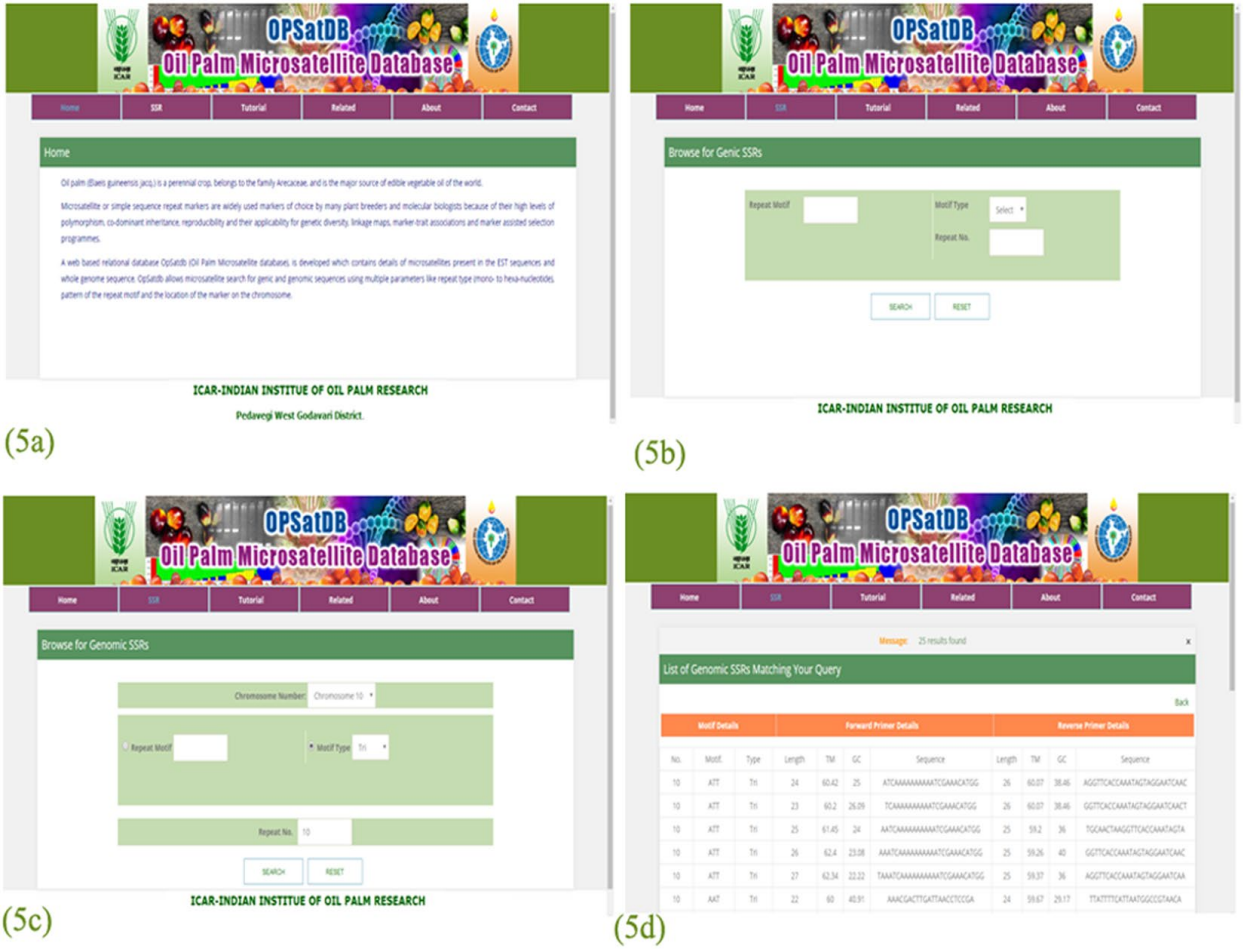
Schematic representation of screen shots of the oil palm microsatellite database (OpSatdb) (the authors acknowledge Director, ICAR-IIOPR for giving permission to publish the website pages).

Molecular markers play very important roles in molecular breeding of crops like oil palm, where marker-assisted selection (MAS) appears to be promising for both major genes and QTLs. Such markers have wider genomics applications in variety identification, studying genetic diversity, linkage map construction and comparative mapping studies. However, until now, no microsatellite database has been available for oil palm. The developed web application OpSatdb (https://ssr.icar.gov.in/index.php) serves as a repository of microsatellite markers, which allows users to trace desired markers. The schematic diagram shows a preview of the database, as well as different search options and a results page (Fig. 3).

## Methods

### Plant genetic resources and DNA extraction

A total of 100 oil palm genetic resources belong to 18 accessions (all are *dura* fruit form) representing four African countries (Zambia, Guinea-Bissau, Tanzania, and Cameroon) were used for validation and genetic diversity analysis of the developed genic and genome-wide SSRs. Characterized 100 oil palm genetic resources belongs 18 accessions with varying numbers of seeds per accession. A list of oil palm genetic resources used in the study, along with accession numbers and origin, is given in Supplementary Table S6. The genomic DNA was extracted using the standard protocol of Murray and Thomson^33^.

### *In silico* mining of genic and whole genome-wide SSRs

Until December 1^st^, 2017, a total of 40, 979 oil palm EST sequences were available in the NCBI website and were downloaded in fasta format for further use. Initially, the poly (A) and poly (T) stretches, which correlated to poly (A) tails, were trimmed at the 5′ or 3′ ends of EST sequences using EST-trimmer software (http://pgrc.ipk-gatersleben.de/misa/download/est_trimmer.pl). The EST sequences of <100 bp were not considered for eSSR identification to preclude the inclusion of low-quality sequences. Then, the non-redundant EST sequences were searched for eSSRs using the online tool websat^34^. The websat software uses primer3 for identification of microsatellite repeats and for primer design. The criteria set for identification of mono- (MNRs), di- (DNRs), tri- (TNRs), tetra- (TeNRs), penta- (PNRs) and hexa-nucleotide repeats (HNRs) were a minimum repeat time of 10, 6, 6, 3, 3, and 3, respectively. The default settings were kept for primer designing. The whole genome sequences were downloaded from the NCBI website chromosome-wise in fasta format. These sequences were further mined for SSRs using MISA (MIcroSAtellite3)^35^. The criteria used for identification of MNRs, DNRs, TNRs, TeNRs, PNRs and HNRs were set to a minimum repeat time of 10, 6, 5, 5, 5, and 5, respectively. Primer pairs were developed using primer3 software.

### Microsatellite marker analysis

The thermal reactions were performed in 25 µL reaction volumes containing about 25–50 ng of template DNA, 2 µL of 10X buffer containing 15 mM MgCl2, 0.2 µM each of forward and reverse primer, 2 µL of 2 mM dNTPs, and 0.2 µL of 1 U of *Taq* DNA polymerase (Invitrogen USA). The amplifications were performed in a Thermo cycler (MJ Research, USA) programmed for an initial denaturation of 3 min at 95°C, followed by 35 cycles of 30 s at 95°C, 30 s of 45°C annealing temperature, and extension of 1.0 min at 72°C, with a final extension of 10 min at 72°C and a final hold at 4°C. The amplicons of PCRs were resolved on 2.5% super fine resolution agarose gels (SFR, Amresco), along with the standard size 100 bp marker at 100 V, and documented in the Bio-Rad gel documentation unit.

### Functional annotation of the EST sequences

The functional annotation of the EST sequences containing SSR primer pairs as analyzed using Blast2GO software^36^. The software performed three main steps: 1) running of the BLASTx, 2) mapping and retrieving the GO terms associated with the blast results, and 3) annotating GO terms for each EST sequence to know their protein function. All of the functions were performed with default settings,; briefly, BLAST was performed at an expectation value of 1.0e-3 and maximum 20 hits, and an HSP length cutoff (default = 33), with low complexity filter was used. The e-value hit filter for mapping was 1.0E^−6^, annotation cut off value was 55, GO weight was 5, and the Hsp-Hit coverage cut off value was 0. The metabolic pathways were generated using KEGG^37^ extension of Blast2GO. The contig sequences were queried for conserved domains using Inter-ProScan^27^, which was an inbuilt program of Blast2GO.

### Validation of genic and genome-wide SSR markers and data analysis for genetic diversity

A random set of developed genic and genome-wide SSR markers was used for their validation on 100 oil palm genetic resources. The 100 oil palm genetic resources belongs 18 accessions with varying numbers of seeds per accession used for genotyping. The genetic diversity and polymorphisms were calculated using Power Marker V3.0 software^38^ for estimating basic statistics viz., PIC value, major and minor allele frequency, gene diversity and heterozygosity. The unweighted pair group method (UPGMA) was used to generate the tree using the shared allele frequency matrix.

### Synteny and conservation between oil palm and rice genomes

The synteny between oil palm and rice genomic sequences was done using the CIRCOS software^32^.

### Design and development of the database OpSatdb

**Figure 4:**
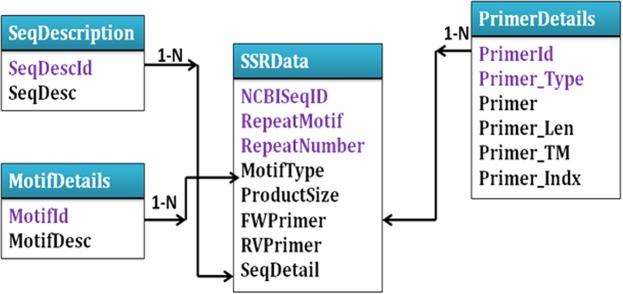
Entity relationship diagram of the oil palm microsatellite database.

The oil palm microsatellite database (https://ssr.icar.gov.in/index.php) is online, freely available for research purposes and non-commercial use, and was developed using MySQL 5.0 (www.mysql.com). The software was designed using the Content Management System (CMS) (Joomla 3.6) for front end design and MySQL as the database, with PHP version 4.5.1 coding for data retrieval. The Entity Relationship (ER) model for the database is given in Fig. 4. The highlighted fields (ncbi seq id, repeat motif, repeat number belonging to SSR data, primer id and primer type belonging to primer details) in the figure are primary keys of character type data. The length, Tm, and GC are of numeric data type. The database provides different search parameters for genic and genome-wide SSRs. The users can search the desired microsatellites using different options such as microsatellite motif, repeat length, and minimum number of repeats, as well as by chromosome. The results will be displayed in a tabulated form having all of the above details, with hyperlinks to primer information.

## Supplementary information

Supplementary material

## Data Availability

The database developed in the present study is available at https://ssr.icar.gov.in/index.php.
