# Supplementary material for: Development and validation of whole genome-wide and genic microsatellite markers in oil palm (Elaeis guineensis Jacq.): First microsatellite database (OpSatdb)
Source: Sci Rep. 2019 Feb 13;9:1899. doi: 10.1038/s41598-018-37737-7 (PMC6374426; doi:10.1038/s41598-018-37737-7)
Supplement: Supplementary file 1 — Supplementary material [file 41598_2018_37737_MOESM1_ESM.pdf]

Development and validation of whole genome-wide and genic microsatellite markers in oil palm (*Elaeis guineensis* Jacq.): First microsatellite database (OpSatdb)

B. Kalyana Babu*#, K. L. Mary Rani#, Sarika Sahu[$]#, R. K. Mathur, P. Naveen Kumar, G. Ravichandran, P. Anitha, and H. P. Bhagya

Supplementary material

Supplementary Figure S1 The percentage distribution of SSR repeat motifs (DNRs, TNRs, TeNRs, PNRs and HNRs) among the oil palm EST sequences

Supplementary Figure S2 The data distribution and Inter-pro scan results of the selected EST sequences of oil palm

Supplementary Figure S3 The species distribution (a) and top hit species distribution (b) results obtained from Blast2GO analysis

Supplementary Table S4 The polymorphism details like allele number, gene diversity, heterozygosity and PIC values generated by using the genic and genome-wide SSRs

Supplementary Table S5 The synteny relationship as obtained from CIRCOS software among the chromosomes of oil palm and rice

Supplementary Table S6 The list of oil palm genetic resources along with their accession name and origin used for validation and genetic diversity analysis using randomly selected each five genic and genome-wide SSR markers.

Supplementary Figure S1

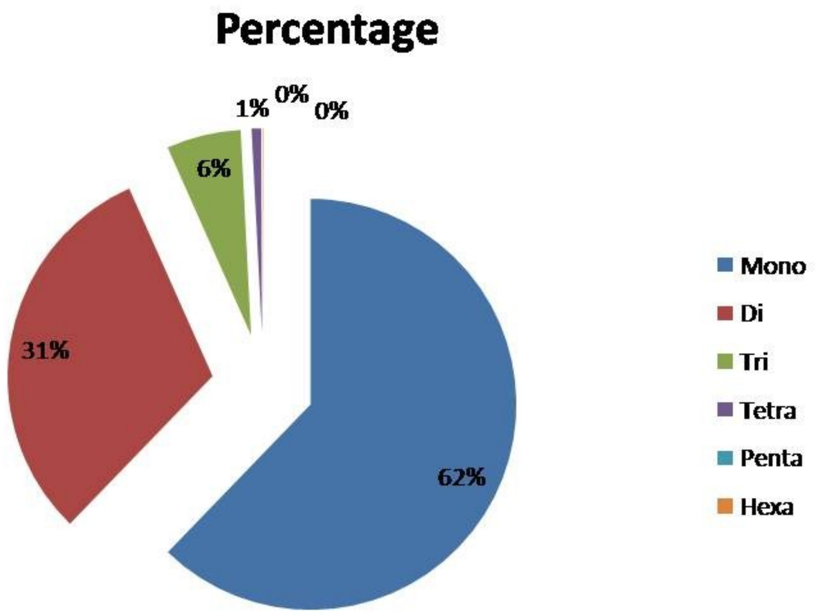

Supplementary Figure S2

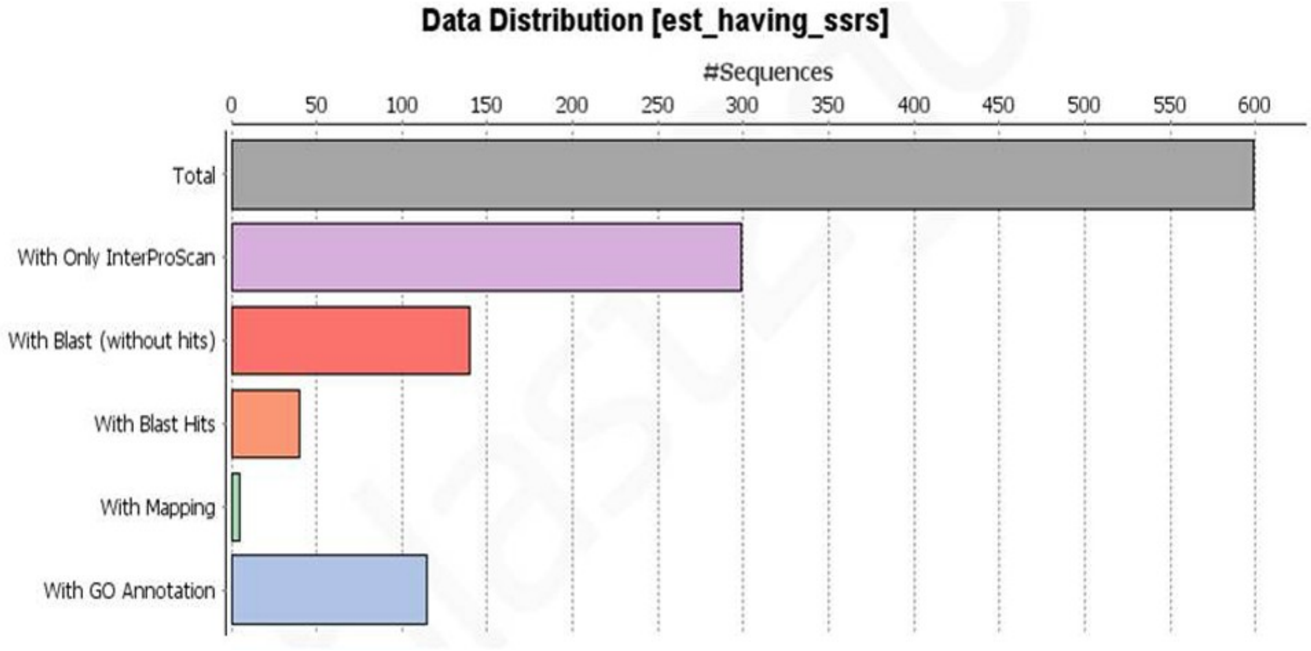

Supplementary Figure S3

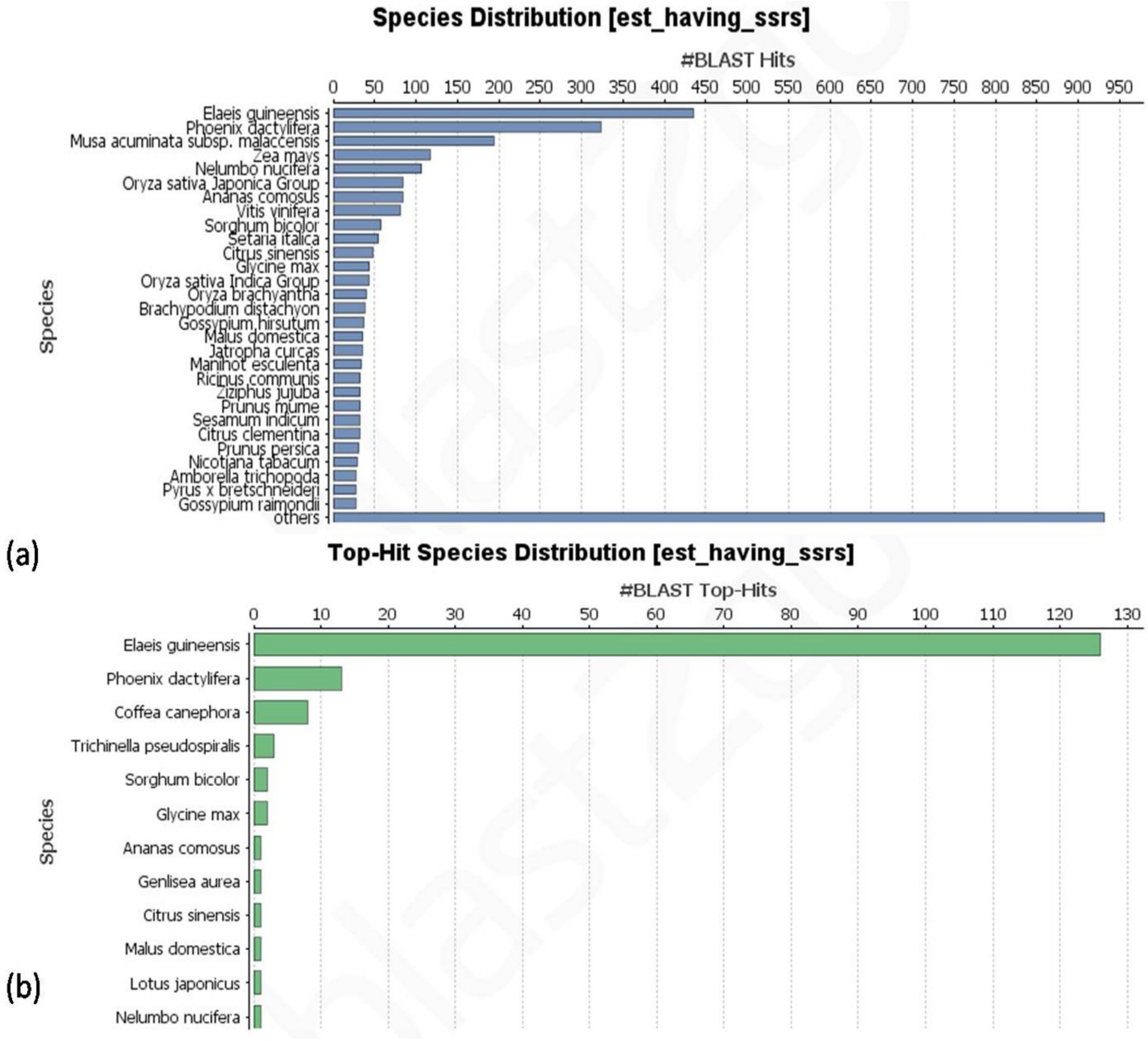

Supplementary Table S4 The polymorphism details like allele number, gene diversity, heterozygosity and PIC values generated by using the genic and genome-wide SSRs

Marker	Allele No	Gene Diversity	Hetero zygosity	PIC
Genic SSRs				
EST17	2	0.46	0.71	0.35
7R2	2	0.49	0.89	0.37
Est12D	2	0.36	0.47	0.29
Est13D	2	0.42	0.60	0.33
Est7R1	2	0.21	0.16	0.19
Mean	2	0.39	0.56	0.30
Genomic SSRs				
pri12	3	0.42	0.38	0.38
PRI16	3	0.57	0.48	0.47
PRI27	3	0.09	0.05	0.09
pri46	4	0.66	0.62	0.59
pri86	3	0.36	0.40	0.33
Mean	3.2	0.42	0.39	0.37

Supplementary Table S5 The synteny relationship as obtained from CIRCOS software among the chromosomes of oil palm and rice

Oil Palm	Rice (Maximum homology)	Rice (less homology)
1	1	3, 12

2	2	4, 5, 6, 8, 9
3	3	1, 2, 6
4	4, 10	2, 3, 7
5	1	3, 5, 11, 12
6	1	3, 5, 7
7	1, 2, 3	-
8	2, 4, 6, 8	9
9	4	1, 2, 11, 12
10	1, 8	2, 3
11	2,3,4,10	-
12	2, 4	6, 10
13	3	7, 8
14	1, 5	3, 7
15	3	4, 8, 10
16	2, 6, 10	8, 9

Supplementary Table S6 The list of oil palm genetic resources along with their accession name and origin used for validation and genetic diversity analysis using randomly selected each five genic and genome-wide SSR markers.

Accession number	Palm number	Origin
CA-12	26	Cameroon
CA-9	27	Cameroon
CA-9	28	Cameroon
TS-11	30	Tanzania
TS-11	31	Tanzania
TS-11	32	Tanzania
TS-11	33	Tanzania
ZS-5	34	Zambia
ZS-5	35	Zambia
ZS-5	36	Zambia
ZS-5	37	Zambia
ZS-8	38	Zambia
ZS-8	39	Zambia
ZS-8	40	Zambia
ZS-8	41	Zambia
ZS-1	44	Zambia
GB-21/310	47	Guinea-Bissau
GB-21/310	48	Guinea-Bissau
GB-21/310	49	Guinea-Bissau
GB-21/310	50	Guinea-Bissau
GB-21/310	55	Guinea-Bissau
ZS-1	56	Zambia
ZS-1	57	Zambia
ZS-1	58	Zambia
ZS-1	59	Zambia
ZS-8	61	Zambia
ZS-5	64	Zambia
ZS-5	65	Zambia
ZS-5	67	Zambia
TS-11	68	Tanzania
TS-11	69	Tanzania
TS-11	70	Tanzania
TS-11	71	Tanzania
CA-11	73	Cameroon
CA-11	74	Cameroon
CA-15	75	Cameroon
CA-15	76	Cameroon
CA-12	77	Cameroon
CA-12	78	Cameroon
CA-16	79	Cameroon
CA-16	80	Cameroon
CA-16	82	Cameroon
ZS-2	83	Zambia
ZS-2	85	Zambia
ZS-2	86	Zambia
TS-9	91	Tanzania

TS-9	92	Tanzania
TS-9	94	Tanzania
ZS-3	99	Zambia
ZS-3	100	Zambia
ZS-3	101	Zambia
ZS-3	102	Zambia
ZS-3	103	Zambia
ZS-3	106	Zambia
ZS-3	107	Zambia
ZS-3	108	Zambia
TS-9	113	Tanzania
TS-9	115	Tanzania
GB-22/311	119	Guinea-Bissau
ZS-2	121	Zambia
ZS-2	122	Zambia
ZS-2	123	Zambia
ZS-2	124	Zambia
CA-17	125	Cameroon
CA-17	126	Cameroon
CA-17	127	Cameroon
CA-17	128	Cameroon
CA-3	129	Cameroon
CA-12	130	Cameroon
CA-12	131	Cameroon
CA-3	133	Cameroon
CA-3	134	Cameroon
CA-3	135	Cameroon
CA-3	136	Cameroon
ZS-1	137	Zambia
ZS-1	138	Zambia
ZS-1	139	Zambia
ZS-1	140	Zambia
TS-11	147	Tanzania
TS-11	148	Tanzania
ZS-8	149	Zambia
ZS-8	150	Zambia
ZS-8	151	Zambia
ZS-8	152	Zambia
ZS-8	163	Zambia
ZS-8	164	Zambia
ZS-8	165	Zambia
ZS-8	166	Zambia
TS-11	168	Tanzania
TS-11	169	Tanzania
TS-11	170	Tanzania
ZS-1	175	Zambia
ZS-1	176	Zambia
ZS-1	177	Zambia

CA-4	179	Cameroon
CA-4	180	Cameroon
CA-4	181	Cameroon
CA-4	182	Cameroon
CA-4	183	Cameroon
CA-12	184	Cameroon
